# How Students Learn during the Pre-Briefing and Observation of Facilitation in a High-Fidelity Patient Simulation: A Narrative Analysis

**DOI:** 10.3390/healthcare12171761

**Published:** 2024-09-04

**Authors:** Florence M. F. Wong

**Affiliations:** School of Nursing, Tung Wah College, Hong Kong SAR, China; florencewong@twc.edu.hk; Tel.: +852-34686838

**Keywords:** high fidelity, simulation, learning, observers, narrative, nursing education

## Abstract

The rapid development of technology has led to the emergence of innovative teaching approaches, such as high-fidelity patient simulation (HFPS). HFPSs have been shown to significantly enhance students’ decision-making and intellectual skills. This study aimed to investigate how students learn from the pre-briefing to observation period of the facilitation of the HFPS based on the original quasi-experimental studies. This study analyzed the narratives from 92 students in the intervention group about their learning during the pre-briefing and observation of facilitation. The results indicated that the students learned more independently, received better support and resources for learning, were provided with more appropriate and safe care for the simulated case, and developed higher-level intellectual skills, such as self-directed learning, critical thinking, and clinical reasoning. Importantly, the structured guidelines provided roles and responsibilities and guiding questions or aspects for observation that directed the students to learn more actively and effectively while performing their roles in the HFPS. The structured guidelines serve as a roadmap to instruct students on learning during pre-briefing and applying what they have learned during the facilitation of the HFPS. This roadmap includes the learning journey from novice to competence in knowledge and skills and also from knowing to application. Therefore, this study’s results have contributed important knowledge about well-structured HFPS guidelines for all stages of the HFPS, addressing the need for adequate guidance and learning support during the pre-briefing and observation of facilitation. The elements identified during the pre-briefing and observation of facilitation are crucial for directing students to learn and significantly enhance their understanding and application of knowledge and skills, ultimately promoting the development of higher-level intellectual skills, professionalism, and engagement. Nurse educators can incorporate these elements into HFPS training in curricula to enhance students’ involvement and optimize the HFPS as an effective teaching tool with structured guidelines providing guidance and support throughout various stages of HFPS training.

## 1. Introduction

The rapid advancement of technology has precipitated a notable shift from traditional to innovative teaching methods [1,2]. The high-fidelity patient simulation (HFPS) has emerged as a popular approach, particularly in healthcare professional training, including nursing [2,3]. In the face of increasingly complex healthcare services, nurses are expected to demonstrate independence and accountability in delivering high-quality patient care [1,2,3]. Accordingly, nursing education must provide students with enhanced learning opportunities to bolster their competence. In conventional nursing education settings, students typically acquire skills in a laboratory setting through low-fidelity patient simulations and tutor-led skill demonstrations.

The HFPS plays a critical role in offering students an immersive learning experience where they can apply their acquired knowledge and skills to simulated patient scenarios tailored to specific conditions and health needs [2,3,4]. Comparing three main simulation sessions, namely, pre-briefing, facilitation (role-playing and observation), and debriefing, the HFPS equips students with valuable hands-on experience and skills [5]. While existing research has demonstrated the positive effects of the HFPS on knowledge acquisition and skill development, the focus has primarily been on student learning during the role-playing and debriefing of the HFPS. Conversely, limited attention has been paid to understanding student learning during the pre-briefing and observation of role-playing activities.

### Background and Literature Review

The HFPS is an innovative teaching methodology that has been widely recognized for its effectiveness and safety in developing students’ abilities by integrating their acquired knowledge and skills in simulated patient scenarios [6,7,8]. HFPS has gained increasing popularity in healthcare professional training programs, including nursing curricula and clinical advancement training initiatives in nursing education [8,9,10]. The Healthcare Simulation Standards of Best Practice (HSSOBP) developed by the International Nursing Association for Clinical Simulation and Learning (INACSL) serves as comprehensive and evidence-based guidelines for the implementation of the HFPS [11,12]. In recent years, numerous studies have utilized these guidelines to assess the outcomes of HFPS training in their intervention groups, offering adequate support and learning opportunities to students. These studies have primarily focused on student learning across three key stages of the HFPS: pre-briefing, facilitation (involving role-playing and observation), and debriefing, highlighting the development of higher-level intellectual skills essential for clinical practice and nursing professionalism, such as problem-solving, clinical reasoning, critical thinking, and managerial skills, which are essential for their clinical practice and nursing professionalism [7,8,9].

Traditionally, students are expected to engage in pre-briefing activities before the HFPS, followed by participation in role-playing sessions in small groups, while the remaining students observe real-time video projections of the role-play. However, students involved in pre-briefing and those observing during the facilitation may be overlooked, potentially leading to passive engagement and diminished motivation to learn [13]. Consequently, students not actively participating in the simulation may lose interest during pre-briefing and observation periods, where they are tasked with observing role-play scenarios and providing feedback during debriefing sessions [14,15]. Yet, there remains a limited understanding of student learning during the pre-briefing phase and while observing their peers’ role-playing activities.

To address this gap, structured HFPS guidelines were developed based on the four HSSOBP standards, namely, pre-briefing, simulation design, facilitation (role-playing and observation), and debriefing in two studies [2,4]. These guidelines offer a systematic approach to guide students in their learning and simulated activities, with promising outcomes demonstrated in enhancing student satisfaction, self-confidence, problem-solving abilities, and critical reasoning skills.

Wong and Wong [2] investigated the impact of these structured HFPS guidelines on 189 undergraduate nursing students through quantitative and narrative methodologies, focusing on student satisfaction (SS) and self-confidence in learning (SCL) throughout the HFPS. The structured HFPS guidelines provide clear direction using learning materials and guiding questions to allow students to learn more appropriately and effectively throughout the HFPS. After the participating students were divided into either the intervention or control group, students in the intervention group received the structured HFPS guidelines that included detailed HFPS instructions, learning materials, guiding questions, and support from the facilitator, while those in the control group received standard treatment with basic HFPS instructions and support from the facilitator. The group allocation was determined based on the selection of the HFPS schedule and the availability of the facilitator who was responsible for either the control or intervention group. The structured guidelines systematically led the entire HFPS and enabled students in the intervention group to be engaged more effectively. This study’s results demonstrated that students in the intervention group, guided by the structured HFPS guidelines, exhibited significant improvements in SS and SCL compared to those in the control group. The narratives from students triangulated their development of learning satisfaction and their enhancement of self-confidence during HFPS training. Similarly, Wong et al. [4] examined problem-solving (PS) and critical reasoning (CR) abilities using the structured HFPS guidelines reporting enhanced skills in students from the intervention group post-HFPS.

Importantly, the structured guidelines were designed not only for the role-playing session of the facilitation and debriefing but also for the pre-briefing and the observation of facilitation. The guidelines included guiding reflective questions to direct students to understand the context through self-study during pre-briefing and observation areas to comment on others’ performance during role-playing. Students’ narratives collected after the HFPS were analyzed to gain insights into their learning experiences during these specific periods, shedding light on the significance of monitoring student learning outcomes throughout HFPSs, including pre-briefing and observation. Therefore, the aim of this study was to explore how students learned through the pre-briefing and observation of facilitation based on their post-HFPS feedback. The results increase the alertness of educators and clinical mentors for HFPSs to pay more attention to student learning outcomes across all stages of the HFPS, including pre-briefing and observation during facilitation, ensuring a comprehensive learning experience for all participants.

## 2. Materials and Methods

### 2.1. Study Design

This study focused on the analysis of the narratives from students who were included in the two primary quasi-experimental studies [2,4] that investigated the effects of structured HFPS guidelines on junior undergraduate nursing students with no prior HFPS or clinical experience. The structured HFPS guidelines included guiding questions for students to engage in learning during the pre-briefing and observation period of the facilitation. Narratives capturing their learning experiences were collected before HFPS and during the observation of facilitation.

### 2.2. Study Aim and Objectives

The study aimed to explore how students learn from the pre-briefing to facilitation stages of HFPS through their narratives, with three specific objectives: (1) to identify students’ learning experiences, (2) to understand their learning methods, and (3) to identify ways to enhance their learning.

### 2.3. Sampling and Study Setting

The study recruited student participants who were from years one and two, aged 18 years or older, with no prior HFPS training or clinical placement experience. The recruitment was conducted through emails to potential student classes between November 2021 and June 2022 at a tertiary healthcare professional training institution in Hong Kong SAR, China. Students were divided in either control or intervention group based on their selected HFPS facilitator’s proposed schedule. Two facilitators were responsible for either the control group or the intervention group at different HFPS training laboratories to ensure consistency of HFPS delivery and prevent treatment contamination.

### 2.4. The Structured HFPS Guidelines

The structured HFPS guidelines, serving as the study framework, along with guiding questions, learning outcomes, and materials related to the simulated patient’s health problems used in this study, offered a structured approach for students to engage in HFPS activities across three major sessions: pre-briefing, facilitation (including observation and role-playing), and debriefing. This study specifically focused on student learning through analysis of their feedback after pre-briefing and observation of facilitation. Only students in the intervention group were required to complete online questions about their learning experiences during the pre-briefing and observation period of the facilitation, while those in the control group received standard support, including information about HFPS, facilitator support, and the case scenario. Both groups had the same intended learning outcomes. Table 1 provides detailed information on the learning resources for these two study periods.

### 2.5. The Instruments

The instruments utilized in this study were open-ended reflective questions, developed by the Principal Investigator (PI) based on her teaching experience in HFPS, the course learning objectives, the teaching materials, and other HFPS resources from two nursing professional training institutions. These instruments underwent review by three experienced nurse educators actively engaged in HFPS training at various nursing professional training institutions. The open-ended reflective questions were designed to elicit responses from students, allowing them to provide valuable feedback following both the pre-briefing and observation periods during facilitation. The specific open-ended reflective questions for these two phases are detailed in Table 2.

### 2.6. Study Procedure

Students in the intervention group (*n* = 92) were grouped into simulation groups comprising eight to ten students for HFPS training, based on their preferred schedule and facilitators’ availability. One week before their HFPS session, students in the intervention group received a comprehensive package of learning materials, while the control group only received the necessary case information. To prevent treatment contamination, two educators with extensive clinical and teaching experience were designated as facilitators for the control group (Facilitator A) and the intervention group (PI acting as Facilitator B).

During the pre-briefing (self-study), students in the intervention group were provided with a package containing learning resources and guiding questions aimed at enhancing their understanding of the simulated case and related care. These guiding questions, developed by the PI, focused on key aspects such as definition, pathophysiology, clinical manifestations, and medical and nursing care for the simulated case. Students were required to respond to these questions online one day before their HFPS session, with encouragement to engage in additional self-study and seek clarification from the facilitator as needed.

On the day of HFPS, the facilitator conducted a briefing to familiarize students with their roles, responsibilities, the environment, and the equipment in the HFPS laboratory. The role-playing HFPS session comprised three scenario sessions, with students divided into smaller groups of three to four students each. Students alternated between participating in the role-playing simulation and observation groups. Those in the observation groups followed the guiding observation areas, aiming at directing them to critically assess the performance of the selected role-playing student through a synchronized real-time video. The guiding areas for observation developed by the PI were to direct students to comment on the selected role-playing student’s performance critically. Those in the observation group were required to provide feedback online on the selected student’s performance based on the guiding areas for observation outlined in Table 3.

Following the role-playing HFPS sessions in three student groups, debriefing sessions were facilitated by the respective group facilitators. However, this article is specifically focused on student learning during the pre-briefing and observation periods of the facilitation. The specific procedures for the remaining HFPS sessions can be referenced in the original articles [2,4].

### 2.7. Ethical Considerations

This study obtained ethical approval from the research committee of the study institution (REC2021102). All students were required to sign an informed consent form before participating in the HFPS and were assigned individual serial numbers for identification when completing online surveys and answering guiding questions at different stages. The surveys used in this study were previously documented in the primary studies [2,4], ensuring data anonymity until analysis. The detailed ethical considerations are described in two primary studies [2,4].

### 2.8. Data Analysis

All reflective answers were independently reviewed by the PI and the research assistant (RA) to uphold the reliability and validity of the findings. Key codes were identified to comprehend student learning during the pre-briefing and observation of facilitation. The PI and RA conducted multiple independent reviews of the students’ narratives to extract accurate and valuable themes. Subsequently, they deliberated on their findings and confirmed the pertinent themes aligning with the study objectives. In case of discrepancies, two independent reviewers engaged in discussions until reaching a consensus.

## 3. Results

### 3.1. Students’ Demographhic Characteristics

The narratives from a total of 92 students in the intervention group were carefully selected for further analysis. The comprehensive demographic characteristics of the students were extensively documented in the primary studies [2,4].

### 3.2. Student Learning at Pre-Briefing and the Period of Being Observers during Facilitation

Upon analyzing the feedback from the 92 students in the intervention group, key factors influencing student learning during the pre-briefing and observation of facilitation were identified. The identified elements are outlined below.

### 3.3. Learning at Pre-Briefing Stage

#### 3.3.1. Adequate Learning Resources to Increase Knowledge Acquisition

Students in the intervention group received a comprehensive package containing detailed instructions on the HFPS, simulation scenarios, and various learning materials related to health issues like arrhythmia and acute myocardial infarction. These resources included information on medical and nursing treatments for the simulated patient, such as 12-lead ECG monitoring, continuous cardiac monitoring, oxygen therapy, and the administration of medication (nitroglycerin, morphine, aspirin, and thrombolytic therapy). While the majority of students were able to read and learn from the materials provided, some found them challenging and sought additional information on the internet. Overall, the students were able to gain valuable knowledge and understanding from the intervention resources.

Student 10: “*When I received the information, I was filled with excitement to dive into reading them. I had high expectations that HFPS training would be a lot of fun. As I started reading, I wasn’t always sure if I fully understood everything, but I definitely learned a lot. The information provided was detailed and presented in a systematic manner, which allowed me to gradually grasp the concepts*”.

Student 38: “*Having a video as part of the resources was really helpful. It enabled me to learn how to perform a 12-lead ECG effectively. Some skills are better understood and learned through visual demonstrations*”.

#### 3.3.2. Clear Instruction and Guidelines

Students found the HFPS instruction and guidelines to be extremely useful. They were provided with clear directions on how to familiarize themselves with the simulated patient and the procedures involved in the HFPS. As a result, they had a clear understanding of what was expected of them during the role-playing session.

Student 7 expressed, “*This simulation training was a completely new experience for me, and I initially felt uncertain about what to do. However, once I read the instruction, everything became clear. It helped me understand the purpose of the simulation and my specific roles within it*”.

Student 33 stated, “*The instruction allowed me to prepare myself mentally for the simulation. I didn’t have much prior knowledge about the case, but the guiding questions provided in the instruction helped me gradually comprehend the health problem. At the very least, I knew that I needed to perform various monitoring tasks to assess the heart and administer specific treatments, such as morphine for chest pain*”.

Student 72 shared, “*When I first received all the information, my initial reaction was ‘no way’. However, when I started reading the instruction and guideline, I found them to be easy to follow. Even though the information was quite difficult, I persevered and kept reading*”.

Student 50 said, “*I read through all the information alongside my classmates who participated in the simulation training with me. We read and discussed everything together, and whenever we didn’t fully understand something, we searched the internet for additional information. Through this collaborative effort, we were able to learn a lot from each other*”.

#### 3.3.3. Adequate Time for Preparation

Most of the students received the learning package one week prior to the HFPS, allowing them ample time for preparation. However, there were some students who were assigned to groups with shorter preparation periods. As a result, students had different perspectives based on the time limits they were given to read the materials.

Student 12 emphasized the importance of time management, stating, “*Having enough time to read the materials gradually, following the provided instructions, was crucial for me. I understood the importance of preparing myself well for this training. I heard that during the simulation, students would be expected to actively participate and provide care based on their knowledge*”.

Student 36 shared, “*I had to manage my own study schedule. With numerous pages to read and only three days to complete them, I could only scan through the materials. It was a bit challenging to absorb all the information in such a short time*”.

Student 55 expressed concern, saying, “*I’m afraid I won’t be able to catch up since I only had two days to prepare. I was instructed to read the information before the simulation, and I regret not starting earlier. I couldn’t complete the online questions, and I hope this won’t cause any embarrassment for me during the training tomorrow*”.

#### 3.3.4. Self-Motivation for Competence Enhancement in Knowledge and Skills

Most students were highly engaged in learning by reading the provided resources to prepare themselves for the HFPS. While Facilitator B would send email reminders, it was ultimately up to the students to determine if they would follow the instructions. Those who did follow the instructions tended to have better time management and showed improved competence in the knowledge and skills of caring for the simulated patient with specific health problems. Such learning was motivated by the students themselves, and it was evident that the more self-motivated they were, the more knowledge and skills they attained.

Student 10 shared, “*I had the option to either take the opportunity to learn or give up. Eventually, I got into it, even though I didn’t understand everything at first. But I kept at it, and I learned that I could do it as long as I didn’t give up and kept pushing myself to learn and understand more about being a nurse*”.

Student 28 shared, “*I started with curiosity about the simulation training and then became fully involved in the training by following the instructions and guidelines. Self-motivation to learn was the driving force that kept me on the learning track*”.

Student 68 stated, “*It’s not easy, but it’s not impossible either. There are no losers, only winners. The more engaged I was, the more I gained. So sometimes, pushing myself a bit more resulted in a surprise*”.

Student 80 expressed, “*I wanted to perform well in front of the tutor. It would have been very embarrassing if I made any silly mistakes. Therefore, I made sure to read all the materials to prepare myself as much as possible*”.

### 3.4. Learning from Observation of HFPS

During the synchronized real-time role-playing of the HFPS, most students were directed to learn through observation. Those in the intervention group were required to follow guiding questions to direct their learning. Students learned through the following elements: clear guiding questions, the application of knowledge and skills from observing the role-players’ performance, and comments on the role-players’ performance.

#### 3.4.1. Performance of Role-Players with Knowledge and Skill Applications

The students learned how to apply their knowledge and skills based on the specific concerns of the simulated patient. They found that the application of knowledge and skills was more challenging than simply acquiring them.

Student 34 shared, “*I realized that providing care for a patient isn’t always a quick process. Before implementing any care, you have to gain the patient’s cooperation. I learned that communication with the patient is crucial, particularly when explaining or performing nursing procedures*”.

Student 68 expressed, “*Through observing the role-playing, I learned that knowledge and skills are crucial in patient care. However, I also realized that respect and understanding are equally important beyond the application of knowledge and skills*”.

#### 3.4.2. Comments on the Performance by Others

Learning from both good deeds and errors is crucial. Students were able to learn from observing the role-players’ actions and mistakes. Commenting is a process of analyzing a situation to determine if it was appropriately treated. Through this critical process, students learned from the good deeds and imitated them, but they also learned from the mistakes to improve their own treatment.

Student 11 expressed, “*I hope that I can perform like one of the role-players. She was able to address the patient’s concerns very well. A nurse should be patient and considerate of the patient’s needs. She knew how to approach the patient*”.

Student 39 identified an error, stating, “*The nasal catheter for oxygen therapy should not be given in that manner. The role-player did not check the patient’s identity before administering the medication. We need to correct this mistake during our turn for role-playing*”.

#### 3.4.3. Discussion with Other Students Who Were Observers

The students were divided into three small groups, and each group had a chance to watch the role-playing session with a different group of students who had already completed their own role-playing session. Through watching the role-playing and discussing with those who had already experienced the simulation, students gained a better understanding of their role-playing experience.

Student 22 shared, “*I completely understood why the role-players were relying on their basic instincts*”.

Student 45 said, “*Those who had already been involved in the role-play session shared that the simulation itself wasn’t difficult, but deciding what to do next and how to do it was the challenging part. This helped me prepare myself emotionally for my own role-playing session*”.

Student 33 expressed excitement about sharing her experience with others, stating, “*Right after our role-playing session, I was eager to share my experience with others. It seemed like we didn’t learn anything while we were in the simulation laboratory. We needed a leader*”.

Student 67 shared what she had learned from others’ experiences, stating, “*They told me that they thought the tutor would tell them what to do, but she allowed them to do whatever they wanted. They also said that the simulated patient would provide some tips or remind them of what to do next*”.

### 3.5. In Both Sessions

#### 3.5.1. Clear Guiding Questions and Observation Areas

Students were provided guiding questions to direct their preparation for and observation during the HFPS role-playing sessions. These questions were specifically related to the performance of the role-players. Each small group of students observed two HFPS role-playing groups, using the same guiding questions.

Student 9 shared, “*The guiding questions were crucial in guiding me to learn in the right direction before simulation training and during the role-playing session*”.

Student 11, “*I was unsure of what I should be doing during the simulation role-playing period until I received the guiding questions. I realized that watching was actually another channel of learning*”.

Student 85 explained how the guiding areas helped focus her observation, stating, “*There were four to five students in the role-playing session. The guiding areas directed me to observe how those students communicated with each other and with the simulated patient, as well as how they implemented care and performed skill procedures. The areas helped me understand what I should be doing during that period*”.

#### 3.5.2. Self-Awareness and Attentiveness

During their watching, students were highly attentive to the role-playing performed by other student groups. They came to realize that what they had learned in the classroom could differ from the actual experience of providing bedside care to a real patient.

Student 9 reflected, “*Providing patient care is not just about implementing a skill procedure. I need to consider other factors that could hinder the procedure, such as sudden changes in the patient’s condition, other patient concerns, and time constraints*”.

Student 21 learned from watching the role-players, stating, “*I realized that I could be just like them, unaware of what I was doing. It made me realize the importance of paying more attention when caring for patients. I need to remain calm and prioritize my actions, always keeping the patient’s well-being as the top priority*”.

Student 36 initially thought that the role-players were unaware of their actions during the simulation. However, this observation taught them the importance of more practice to improve competence in providing appropriate care and managing different conditions effectively.

#### 3.5.3. Active Self-Engagement

Students emphasized the importance of their self-engagement in all simulation sessions. Their active participation enabled them to explore more learning opportunities, not only for knowledge enhancement but also for the development of high-intellectual skills such as problem-solving, clinical reasoning, and critical-thinking abilities throughout the entire HFPS, including the pre-briefing and observation of facilitation. Additionally, students found that their active engagement in learning facilitated their learning in future HFPS sessions.

Student 22 shared, “*Initially, I only wanted to learn about the simulation. When I received the package, I wasn’t too keen on reading the materials. However, once I got involved, I learned about not only the simulation but also many other things that a nurse should learn*”.

Student 92 expressed, “*I felt like I had a significant knowledge deficit at first, but it was interesting to understand the cardiac and 12-lead ECG. I searched for additional information about ECG and caring for cardiac patients. When I could not answer the guiding questions, I critically thought about the answers and tried to find them through various internet resources. This information helped me analyze the simulated case and understand the purpose of treatment*”.

Student 22 also reflected on their observation of the role-players, stating, “*I did not know what they were doing, but I imagined what I would do if I were in their position. I’m sure they had thought before they acted. While watching them, I also had a lot of thinking to do*”.

Finally, student 77 noted the importance of active engagement in learning, stating, “*I kept watching the role-playing because I wanted to learn from them. I realized that I could not understand unless I involved myself in the process*”.

In summary, students can learn effectively during the pre-briefing and observation of facilitation while they are observers. The learning elements that enable students to learn more effectively during the pre-briefing session include adequate learning resources, clear instruction and guidelines, adequate time for preparation, and self-motivation for competence enhancement in knowledge and skills. During the facilitation session, students learn effectively as observers through the performance of role-players with knowledge and skill applications, comments on the performance by others, and discussion with other students who were observers. In addition, clear guiding questions, adequate time for preparation, self-awareness, attentiveness, self-motivation for competence enhancement in knowledge and skills, and active self-engagement in both sessions are crucial elements for students to learn effectively during these two specific sessions of the HFPS. These learning elements are the interacting areas or strategies to enhance students’ competence in personal and professional development.

## 4. Discussion

This study aimed to explore the elements of student learning during the pre-briefing session and the observation period of the facilitation session, based on students’ reflections from open-ended reflective questions. It is crucial to emphasize that students derive significant benefits in terms of knowledge and skills, encompassing practical skills and high intellectual skills, contributing to their personal and professional development through the pre-briefing and observation of facilitation. The elements identified in pre-briefing are crucial in preparing and nurturing students’ competence in knowledge acquisition and skills with appropriate support and resources while those identified in the observation of facilitation are crucial for the applications and practices that students have learned for the realism of professionalism. All elements identified for pre-briefing and observation are essential to help direct students’ learning and develop their learning attributes.

The pre-briefing is vital in enabling students to practice effectively for the HFPS. Initially, students are expected to grasp the ground rules, simulation content, and their roles and responsibilities [11]. This study revealed that the HFPS structured guidelines included instructions, ground rules, and a package of learning materials to foster students’ understanding and involvement to better prepare students for the HFPS. The structured HFPS guidelines provide systematic direction to guide students’ learning more effectively and enhance their understanding and competence in providing care. Clear instructions are essential in helping students comprehend HFPS arrangements, their roles and responsibilities of students, and their expectations in each simulated scenario session. This clarity boosts student cooperation and motivation to follow steps and systematically reach the achievement of learning outcomes [12]. Clear instructions enhance student engagement and aid in achieving their learning expectations throughout the HFPS.

The provision of appropriate and easily understandable learning resources enhances students’ willingness to engage in learning for the HFPS, offering stimulation and guidance for self-study. Students actively involved in pre-briefing can effectively apply their knowledge and skills. They can also effectively provide feedback on selected student performance during role-play when acting as observers. Successful engagement leads to increased satisfaction, confidence, motivation, and willingness to learn. Therefore, being well prepared during the pre-briefing session, aimed at performance-driven outcomes, significantly boosts student engagement, self-confidence, and satisfaction in learning during the observation and role-playing periods in the facilitation of the HFPS [2,16,17].

In addition to favorable structured guidelines, adequate time and appropriate learning materials are essential for student engagement in learning. A well-executed pre-briefing enables student success and improves their understanding and performance during facilitation, whether as observers or role-players, and in debriefing for self-reflection after the HFPS [16,17,18]. Students are encouraged to engage in self-study on unfamiliar content during pre-briefing, requiring active learning using provided resources to enhance understanding. To cater to students’ comprehension levels, it is crucial to offer suitable, engaging, and easily digestible learning materials to facilitate student learning [14]. Active engagement is crucial for students to benefit from self-study and peer interaction, gaining insights from various learning perspectives [19,20,21]. Increased engagement leads to surpassing expected outcomes, as motivated students pursue further study [22].

Healthcare students need to develop self-directed study skills as their learning attitudes and interests intensify [22]. Self-directed learning is bolstered by the heightened self-awareness of competence inadequacy [23,24], self-attentiveness [3,25], and self-motivation [13,14,15]. Motivation drives self-directed learning and critical thinking, promoting continuous learning as needed [5,13,26]. Strong self-motivation aids in knowledge acquisition, higher intellectual skill development (critical thinking, problem-solving, analytical skills), self-reflection, and personal and professional growth [15]. This self-awareness increases attentiveness, motivating students to learn until they feel satisfied and achieve their learning goals, culminating in successful learning [23,24,25,26]. To cultivate students’ self-directed learning and enhance their engagement of learning through the HFPS, it is essential to stimulate students’ learning interest. Once their learning interest is piqued, students will seek further study through surfing online resources to fill knowledge gaps [3,23,24,25], further fueling self-motivation that enhances competency in knowledge and professional development [15,26]. In this study, students were encouraged to engage in self-directed learning and seek guidance from facilitators to enhance their understanding and competence [2,5].

As a matter of fact, the innovative HFPS itself ignites students’ learning interest, motivating them to delve deeper into knowledge acquisition and skill advancement. The HFPS plays a pivotal role in providing students with opportunities to apply and refine their knowledge and skills in simulated patient scenarios [2,4]. The facilitation session in the HFPS is a crucial avenue for students to experience and implement their knowledge and skills under supervision and support to achieve desired learning outcomes [12].

While many studies on the HFPS focus primarily on students engaged in the role-playing, the roles of observers are often overlooked. A scoping review exploring the roles of observers in simulation among undergraduate nursing students revealed that observation can enhance reflective capacity and foster higher-intellectual skills, such as critical thinking and clinical reasoning [13]. Through observation, students can improve their practice identifying gaps in their knowledge, learning from their peers, and broadening their perspectives through discussions and knowledge exchange [20,27]. During observation, students increase individual student learning through empowering and inspiring from others’ perspectives [5,22,28]. They may also learn through discussion with each other, exchanging ideas, and broadening knowledge. Therefore, the observation period serves as an effective means for self-reflection and peer improvement, involving feedback, mutual discussions, and peer evaluations [29,30,31]. By sharing ideas and experiences, students can generate creative insights, fostering mutual respect, communication, self-awareness, and self-reflection, not only for those actively participating in role-playing but also for student observers [12,32].

It is imperative to increase students’ active participation to heighten their awareness of roles and responsibilities, ensuring they have defined tasks as observers instead of being overlooked [20]. In this study, the structured HFPS guidelines provide directions for students to engage effectively during observation by providing areas of focus to enhance their understanding of the simulation progress [13]. While observing, students learn from role-players how to communicate with the simulated patient, formulate care plans, prioritize their skill implementation, and embody the role of nurses in a simulated clinical environment. Through observation, students analyze and comment on role-players’ performances, enhancing higher-intellectual skills, such as problem-solving, critical thinking, and analytic skill abilities. Importantly, student observers can identify blind-spots that role-players may overlook, providing feedback for the improvement and prevention of malpractice and medical errors. The HFPS allows for errors as a valuable learning tool [13,30]. Student observers benefit from learning through both correct and incorrect performances by their peers in the HFPS, without the pressure of role-playing, leading to enhanced learning experiences [29,33].

In summary, this study delved into the elements of student learning during the pre-briefing and observation of facilitation in the HFPS through students’ reflections The pre-briefing was highlighted as crucial for preparing students by providing structured guidelines, clear instructions, and appropriate learning resources to enhance their understanding and engagement in the HFPS. Active involvement in pre-briefing facilitated knowledge acquisition and skill application, leading to increased satisfaction, confidence, and motivation in learning. Additionally, the observation period during facilitation allowed students to learn from their peers, improve critical thinking and clinical reasoning skills, and engage in self-reflection and peer evaluation. The study emphasized the importance of fostering active participation and providing guidance for student observers to enhance their learning experience and contribute to their overall professional development.

### 4.1. Practical Recommendations

This study’s results highlight that the implementation of well-structured HFPS guidelines can significantly impact student learning in terms of knowledge and skills during each HFPS session. Research has demonstrated that the HFPS enhances student learning, promoting cognitive engagement, fostering clinical reasoning, and facilitating effective decision-making [34]. By incorporating students’ perspectives into their learning process, the HFPS can optimize learning outcomes [2,32]. The structured HFPS guidelines should provide clear directions, essential information on rules, regulations, students’ roles and responsibilities, and learning objectives to actively engage students and enhance their professional competence [5]. Utilizing open-ended reflective guiding questions can offer systematic and concise guidance to students, promoting effective learning engagement, knowledge expansion, and skill development [2,35].

In healthcare education, including nursing, the HFPS has proven to be a valuable tool for student learning. Enhancing resources and support for student learning is essential for HFPS training. The structured HFPS guidelines serve as a roadmap for students to progress from understanding theoretical knowledge and skills to applying them effectively in HFPS scenarios. The pre-briefing and observation periods are critical learning stages managed entirely by students, emphasizing the need for adequate resources and support to motivate student learning. It is essential for nurse educators and clinical mentors involved in HFPS training to provide guidance and support to students during these key stages, ensuring they acquire the necessary theoretical knowledge and practical skills to deliver safe and appropriate nursing care to patients [2,4,6]. By enhancing students’ exposure and learning opportunities during the pre-briefing and observation periods, their learning outcomes can be significantly improved.

### 4.2. Strengths and Limitations

This study exhibits several strengths, including the use of narrative analysis, which provided valuable insights into how students engage in learning during the pre-briefing and observation of facilitation in the HFPS. The narrative analysis approach allowed for a deep exploration of students’ experiences and perspectives, shedding light on their learning processes and outcomes.

However, the generalizability of the results is limited due to the recruitment of participants from a single professional training institution. To enhance the external validity of the results, future research should aim to replicate the study at multiple centers to capture a more diverse range of student experiences and perspectives. By conducting similar studies across various institutions, the findings can be more broadly applicable and representative of a larger student population. Another limitation is the absence of narratives from students in the control group, which hinders the comparison of learning outcomes between the intervention and control groups. To address this gap in understanding, future studies should incorporate narratives from both groups during the pre-briefing and observation sessions of the HFPS. A comparative analysis of critical thinking skills and competence levels between the intervention and control groups would provide valuable insights into the effectiveness of the HFPS training approach.

At last, it is recommended that feedback be provided to students after they submit their responses to the guiding questions online following the pre-briefing session. This feedback mechanism can facilitate student improvement, clarify misunderstandings, and enhance the learning experience. Incorporating feedback loops into the study design can promote continuous learning and development among students, contributing to a more effective educational process.

## 5. Conclusions

This study underscores the significance of student learning during the pre-briefing and observation period of facilitate in the HFPS. The structured HFPS guidelines serve as a roadmap, guiding students through the learning process from preparation in the pre-briefing stage to the practical application of acquired knowledge and skills during the facilitation of the HFPS. This roadmap facilitates students’ progression from novice to competence in both theoretical knowledge and practical skills, transitioning from understanding concepts to their real-world application. These guidelines direct students to actively engage in self-directed study, participate in group discussions, and observe role-playing simulations, fostering a holistic learning experience. As the HFPS has emerged as an effective teaching and learning method in healthcare professional training, the identified elements are essential to enhance and motivate student learning during the HFPS. During pre-briefing, students are tasked with preparing themselves to develop and refine their knowledge and skills for competency in practice. The identified elements for both the pre-briefing and observation period of the facilitation are essential in optimizing student learning during these two specific sessions, relying heavily on student participation and engagement. Given the widespread use of the HFPS in nurse education and clinical training, nurse educators and clinical mentors can incorporate these elements into HFPS training curricula and programs to enhance participant engagement and optimize the HFPS as an effective teaching tool. By providing structured guidelines to offer guidance and support across various stages of HFPS training, educators can ensure a comprehensive and enriching learning experience for students.

## Figures and Tables

**Table 1:** Details of the learning resources received by two groups for two study periods between two groups.

Study Period	Control (by Facilitator A)		Intervention (by Facilitator B)	
	Learning objectives	Learning resources	Learning objectives	Learning resources
Pre-briefing	Course intended learning outcomes	Case scenario Students were informed to seek assistance from Facilitator A if needed	Course intended learning outcomes After reading the learning materials, students are able to understand more about a major health problem in terms of its pathophysiology, precipitating factors, clinical manifestations, medical and nursing care, and complications	Case scenario Structured HFPS guidelines Journal papers related to the case and respective management PowerPoint presentation with audio-recording One page of keynotes A set of guiding questions for reparation and self-learning A set of questions for post-study reflection Facilitator B served as a supporter and will encourage student participants to prepare for the HFPS using provided learning resources and further self-study via emails
Observation period	Not specific	Synchronized real-time role-play	Students were able to learn through observing the role-playing HFPS performed by other student groups	Synchronized real-time role-play A set of questions related to the role-playing session They were encouraged to answer or write their comments and reflection of their learning specifically during this period

**Table 2:** Details of the open-ended reflective questions used after pre-briefing and observation of facilitation.

Period	Questions
Post- pre-briefing	What are your thoughts on the learning materials provided for your learning? What do you feel about the amount and appropriateness of the learning materials for your learning needs? In what ways did the learning materials benefit your learning? How did you go about enhancing your learning or improving your understanding of the health topic? In addition to the provided learning materials, did you utilize other methods to aid in your learning prior to engaging in HFPS?
Post-role-play observation	According to your observation of the role-player during the HFPS, please provide answers to the following questions: How did you maintain your focus while watching the role-play? What did you learn from observing the role-play of another group? How do you believe you can enhance your learning through observing role-plays?

**Table 3:** Details of the guiding areas for observation used during observation of facilitation.

Period	Observations
During the role-playing	Select a player; observe and comment his/her performance in the following areas for observation. Ensuring the simulated patient receives sufficient assessment and monitoring. Addressing the concerns expressed by the simulated patient. Providing appropriate and safe care for the simulated patient. Carrying out nursing care using the correct procedures. Using additional skills such as communication, critical thinking, and clinical reasoning abilities.

## Data Availability

The data presented in this study are available on request from the corresponding author. The data are not publicly available to maintain confidentiality.
